# Gestational Diabetes Mellitus and its Effects on the Developing Cerebellum: A Narrative Review on Experimental Studies

**DOI:** 10.22037/IJCN.V18I2.36632

**Published:** 2024-03-12

**Authors:** Mohammad Javad SAEEDI BORUJENI, Pilar CODONER FRANCH, Eulalia ALONSO IGLESIAS, Marie GOMBERT

**Affiliations:** 1 Department of Medical Basic Sciences, Islamic Azad University, Isfahan (Khorasgan) Branch, Isfahan, Iran; 2 Department of Pediatrics, Obstetrics and Gynecology, University of Valencia, Valencia, Spain; 3 Department of Biochemistry and Molecular Biology, University of Valencia, Valencia, Spain

**Keywords:** Gestational Diabetes Mellitus, Neurotoxin Agents, Developing Cerebellum

## Abstract

Diabetes mellitus during pregnancy is a common complication of gestation, but its effects on the offspring’s development are poorly understood. Recently, some studies reported that gestational diabetes mellitus (GDM) impairs cerebellar development, and some genetic alterations have been described as consequences. Cerebellum, one of the hindbrain derived structures in the posterior cranial fossa, plays a crucial role in cognition and behavioral functions. In recent years, some surveys stated that gestational diabetes has adverse effects on the fetus’s cerebellum. Disruption of cerebellar cortex morphogenesis, reduce the volume of the cerebellum, reduce the thickness of cerebellar cortex layers, and its neuronal cells and effects on the expression of synaptophysin, insulin, and insulin-like growth factor -1 receptors are some of the maternal diabetes effects on developing cerebellum. On other hand, GDM, as a neurotoxic agent, impaired cerebellar development and could be a cause for the behavioral, functional, and structural anomalies observed in pups of diabetic mothers. Based on the literature review, most studies have pointed out that administering insulin in patients with GDM decreased the cellular and molecular alterations that induced by GDM in the developing cerebellum. Undoubtedly, screening strategies for all pregnant women are necessary.

## Introduction

Gestational diabetes mellitus (GDM) stands as the most prevalent health complication during pregnancy, and the number of young women with undiagnosed GDM is rising globally. While the precise origin of GDM remains unclear, several factors are believed to contribute significantly to its development. These include maternal overweight and obesity, a prior history of GDM, advanced maternal age during pregnancy, Type 2 diabetes mellitus, and specific ethnic backgrounds (1-3). Disturbed glucose hemostasis with beginning or first recognition during gestation is the main feature of GDM (4). This type of diabetes mellitus (DM) is described by insufficient pancreatic beta-cell performance to meet the increased insulin need due to pregnancy. Hyperglycemia, autoimmune diseases, monogenic causes, and chronic insulin resistance are some of the proposed factors associated with insufficient beta-cell function in GDM (4-6). In GDM, glucose, as a leading source of brain energy, is transported across the placental tissue utilizing facilitated diffusion. The results of the condition, as mentioned earlier, are hyperinsulinemia in the fetus, accelerated fetal growth, and possible neonatal hypoglycemia at delivery. The consequences and adverse effects of GDM on embryos may extend beyond the childhood period, but the issue is open to discussion (6-10).

GDM impairs the development of the Central Nervous System (CNS) in offspring, and learning disabilities, memory weakness, impaired language development, motor problems, and antisocial behaviors are some of the unwanted GDM consequences (6, 11-14). So far, many clinical studies investigated the performance of children born to mothers with GDM. Their surveys presented a negative correlation between the performance of these children on various cognition and behavioral tests (12, 15-17). GDM-induced fetal encephalopathy is a complex cascade of events involving cellular and molecular alterations related to neurogenesis, neuronal migration, differentiation, and cell survival (18-21). In recent years, investigation of GDM’s effects on the expression of brain regulatory genes that control brain development has been considered. Based on previous surveys, alterations of involved genes in CNS development are the basis of neurodevelopmental and neurobehavioral abnormalities observed in children of diabetic mothers. Apoptosis is a form of cell death occurring in some physiological and non-physiological conditions (22). Many studies have shown experimental models of GDM-induced neuronal apoptosis in the CNS of offspring (23). 

The cerebellum is one of the hindbrain-derived structures in the posterior cranial fossa involved in the coordination and control of voluntary movements (24). Moreover, the cerebellum plays a crucial role in cognition and behavioral functions (25-27). Cerebellar involvement has been reported in some psychiatric disorders such as autism (28), attention deficit hyperactivity disorder (ADHD) (29, 30), dyslexia (29) and schizophrenia(31, 32). Recent studies have indicated that GDM can hinder cerebellum development, and genetic alterations have been identified as consequences of this condition. So far, limited studies have investigated the effect of GDM on the development of the fetal cerebellum, and most of them are in the category of experimental and animal studies. The present review summarizes the recent findings about maternal diabetes and its effects on the developing cerebellum.

**Literature review**

Many clinical and experimental studies have been conducted on the effect of GDM on developing the fetus and the occurrence of late-term disorders in the newborn. However, the studies that have explicitly been conducted on developing structures related to the nervous system, such as the cerebellum, hippocampus, and the like, are scarce and mostly limited to animal studies. In order to review of the literature, a search was made in Google Scholar, PubMed, ScienceDirect, and the like, with the keywords GDM, cerebellum, development of CNS and nervous system defects, and articles related to the subject, i.e., the effects of GDM on the development of the nervous system and cerebellum were extracted. 

**Overview of gestational diabetes and molecular aspects**

One of the central representations of pregnancy is a decrease in insulin sensitivity. This condition compensates with increased insulin secretion (4, 33). Diabetes during pregnancy occurs in subjects who are unable to compensate for insulin insensitivity with adequate insulin secretion and who have never had a positive history of DM prior to gestation. However, some surveys suggest the presence of susceptibilities prior to the disease, such as genetic mutations associated with energy homeostasis (34-36). A positive history of GDM in prior pregnancy is one of the main reported risk factors that increase the risk of diabetes in a subsequent pregnancy. Furthermore, some surveys reported a significant relation between increased maternal age and increased occurrence of GDM. Family history of DM, high body mass index (BMI), non-white ethnic background, polycystic ovary syndrome, smoking and gene polymorphism are some of the other proposed risk factors of GDM (37-39). The above factors are non-specific, and roughly 40–60% of pregnant women with DM have no noticeable risk factor; for this reason, many advocate screening all women (40). Usually, GDM exhibits no symptoms in women, but in some cases, it may exhibit increased thirst, increased urination, vomiting and nausea(41). DM during pregnancy increases the risk of short-and long-term complications for the offspring. Hyperinsulinemia/hypoglycemia, hyperbilirubinemia, Macrosomia and shoulder dystocia are some of the short term complications in offspring that are exposed to GDM (42-45). Increased risk of occurrence of DM, obesity and cardiovascular disease are some of the common long-term complications in neonates that are born to mothers with GDM (7, 8, 44-46). Routine treatments of DM during pregnancy are effective in preventing short-term complications in neonates, but it remains uncertain whether long-term complications can be prevented. 

Recently, some studies proposed the crucial role of microRNA in the pathogenesis of GDM (47, 48). MicroRNAs are a class of non-coding RNAs known as epigenetic regulators (49-53). The placental tissue creates some types of microRNA that are released into the mother’s circulation. During pregnancy, the microRNA mentioned above genes are encoded into particular clusters and expressed specifically by placental tissue in a time-dependent manner (54). For the first time, Zhao et al. reported significant downregulations of miR-132, miR-29a, and miR-222 in the plasma of pregnant women who suffered from GDM (55). The microRNA above is associated with sufficient beta cell function, IS and glucose homeostasis(56, 57). The study by Shi et al. reported the upregulation of miR-222 in the omental tissue of GDM subjects (58). Other findings of their survey were a positive correlation of miR-222 levels with a concentration of maternal serum estradiol (increased in the GDM’s subjects) and a negative correlation with protein concentration of glucose transporter 4 (GLUT4) and estrogen receptor in the maternal omental tissue (58). The microRNAs derived from the placental tissue were released into maternal blood circulation and carried into plasma by exosomes (59-61). The exosomes containing microRNAs are released into mother circulation in the sixth of pregnancy and marked with the specific placental alkaline phosphatase enzyme, making their isolation and consequent representation possible during gestation(62). After Zhao et al.’s report, many studies aimed to analyze the microRNA alterations in GDM patients. So far, dysregulation of miR-518d, miR-101, miR-16-5p, miR-17-5p, miR-19a-3p, miR-19b-3p and miR-20a-5p were reported following the occurrence of GDM (54). 

As previously mentioned, genetic susceptibility plays a crucial role in GDM. The significantly higher occurrence of type 2 DM in subjects with a positive history of GDM, family history of type 2 DM and incidence of GDM and similarity in microRNA associated with type 2 DM and GDM raise the question to what degree of the genetic planning of Type 2 of DM and GDM are similar (36, 63, 64). Recently, some candidate genes were proposed associated with DM during pregnancy. TCF7L2, GCK, KCNJ11, KCNQ1, CDKAL1, IGF2BP2, MTNR1B, and IRS1 are related genes with GDM. The exciting topic is the similarity of the genes mentioned above in the pathogenesis of type 2 DM and GDM(36). 

**Gestational diabetes mellitus and central nervous birth defects**

As previously mentioned, infant’s anomalies rates are significantly higher in neonates born to GDM mothers. The time of GDM onset and severity plays a critical role in the maldevelopment of the fetus. So far, the precise mechanisms associated with GDM’s effects on developing growing fetuses are not fully understood ; on the other hand, there are many ambiguous aspects about the mother’s diabetes effects on her child (65, 66). Some cellular and molecular alterations about the GDM effects on a fetus’s CNS have been proposed. The alterations above are associated with changes in patterns of development of neuronal integrity, synaptic membranes, neurotransmitter systems and neuronal growth factors expression (13, 23, 67-69). In this part, we summarize some of the proposed reasons involved in CNS pathogenesis following GDM.

Because of a disturbance in energy homeostasis, GDM is associated with fatty acid metabolism dysregulation (70). In this condition, the concentration of docosahexaenoic acid (DHA) in umbilical venous blood was reduced; this reduction of DHA reflects lower transportation of the aforementioned acid to the fetus (71, 72). DHA is the most abundant fatty acid in the brain and retina, so DHA includes 40% of the polyunsaturated fatty acids in the brain and 60% of the acids mentioned above in the retina. Furthermore, roughly half the weight of the neuron’s plasma membrane is composed of DHA (73-75). In prenatal life, DHA plays a crucial role in neurogenesis, neurotransmission and protection of CNS from oxidative stress(76, 77). Due to GDM, the availability of this acid is reduced in the fetus’s CNS and induces maldevelopment of the nervous system.

One of the adverse effects of GDM is the increased oxidative stress. Some animal studies indicated that oxygen radicals play a critical role in some neurodevelopmental processes, such as neuronal differentiation, neuroplasticity and timing of neuronal formation. Hence, any alterations or imbalances in the aforementioned signals can lead to the maldevelopment of the fetus’s CNS (78-80). In increased oxidative stress, free radicals inactivate some vital functions of the lipids and protein molecules, possibly leading to cell death. One of the other findings that proved the role of oxidative stress in central nervous birth defects in infants born to diabetic mothers is a significant reduction of anomalies mentioned above using antioxidant therapy (79). Based on the above information, oxidative stress is related to the pathophysiology of the fetal mal-development of the nervous system.

One of the other factors involved in diabetic neuropathies is inflammatory factors. Demonstratively, GDM increased the concentration of inflammatory molecules, and this condition is related to the maldevelopment of the fetus’s CNS. Tumor necrosis factor-alpha (TNF-alpha) and interleukin-6 (IL6) are two main proposed inflammatory factors that increase in infants born to diabetic mothers and have been concerned with neuronal impairment. Based on the results of some studies, the molecular immune changes that occur during fetal life may persist into the childhood period, so increased inflammatory factors formed by induction of GDM may be associated with poor mental performance of children born to diabetic mothers (81-86). 

Insulin-like growth factor-1 (IGF-1) is a factor similar to the molecular architect of insulin. IGF-1 plays a crucial role in fetal development and continues to have anabolic effects in postnatal life. Both insulin and IGF-1 belong to the insulin superfamily and are involved in the normal development of CNS. Proposedly, the superfamily mentioned above influences many biological functions of CNS development, such as neuronal and glial cells proliferation, neuronal differentiation, neuronal reformation, and synaptogenesis. Insulin and IGF-1 apply their effects through binding to their receptor (insulin receptor and IGF-1 receptor) (87-89). Some surveys indicate defects in the receptors expression in the fetus’s CNS following GDM, as mentioned above (90). 

**Gestational diabetes mellitus and developing cerebellum**

One part of the rat’s brains that experience morphogenetic alterations after birth is the cerebellum (91). The precise equivalence in age between rats and the human brain is not apparent, but proposedly, the neonatal period in rats matched the third trimester of pregnancy in humans (92). The cerebellar cortex is a dynamic structure in which neuronal proliferation, migration, and granule cell differentiation occur (93). Regarding embryology, roughly during the first week after birth, neuronal generation in the external granule layer of the cerebellar cortex was performed. Then, after rapid cell proliferation in the layer mentioned above , numerous granule cells were formed that migrate with the help of radial glial cells through the molecular layer to reach their destination within the internal granule layer and differentiate into mature cerebellar neurons (91, 94). Unlike granular cells, all of the purkinje cells of the cerebellar cortex are generated before birth on embryonic days thirteen to sixteen, and dendritic outgrowth of them and synaptogenesis occur during the first weeks after birth (95). Any alteration in the above critical period may affect the cerebellar cortex development. Delayed cerebellar cortex development has been observed after prenatal exposure to GDM (90, 96, 97). 

Hami et al. investigated the effects of maternal diabetes on stereological parameters of the developing cerebellum of a rat’s fetus (96). In their study, they analyzed the GDM impacts on cerebellar volume, the thickness, and the number of cells in the different layers of the cerebellar cortex on postnatal days 0, 7, and 14. The study’s results mentioned earlier indicated that GDM disrupted the cerebellar cortex morphogenesis. Based on their study, the cerebellar volume and the thickness of all three layers of the cerebellar cortex, including internal granule, molecular, and external granule layers, were reduced significantly. Moreover, the densities of granular and purkinje cells were increased at postnatal day 0. The other finding from their study was a reduction of granular and purkinje cell numbers in the cerebellar cortex of neonates born to diabetic mothers at postnatal days 7 and 14 (96). In another study, Mirarab Razi et al. surveyed the effects of GDM on the neuronal cells of neonates’ cerebellum (98, 99). Their study performed on day 7 of postnatal life was agreed with the study done by Hami et al. previously mentioned. 

As stated in the previous paragraph, insulin superfamily members (specifically insulin and IGF-1) play a crucial role in brain development during prenatal life. Haghir et al., in an animal study, examined the effects of diabetes mellitus during pregnancy on the expression of insulin and IGF-1 receptors in the developing cerebellum of rat neonates(90). Their investigation on postnatal days 0, 7, and 14 showed that GDM powerfully influences insulin and IGF-1 receptors in the developing cerebellum. Another significant finding from their study was the neuroprotective effects of administered insulin on regulating receptors mentioned above expression (90). Taken together, based on the above-mentioned study, it can be concluded that maternal hyperglycemia and neonatal hyperinsulinemia may disrupt the development of offspring’s cerebelli by dysregulation of expression of the insulin and IGF-1 receptors in both at mRNA and protein levels. In the most recent study, Hami et al. investigated the difference in the distribution of IGF-1 receptors in the developing cerebellar cortex of rats born to diabetic mothers (97). Their study showed that GDM markedly influences the localization of IGF-1 receptors in the developing cortex of the cerebellum. They stated that GDM, as a neurotoxic agent, impaired cerebellar development and could be a cause for the structural and functional anomalies observed in pups of diabetic mothers. 

Synaptophysin, or significant synaptic vesicle protein p38, is one of the crucial proteins involved in the normal development of CNS, and its presence is associated with synaptogenesis and synaptic density (100, 101). This protein was one of the first recognized proteins in the synaptic vesicle, accounting for roughly seven to ten percent of whole synaptic vesicle proteins. In an excellent experimental study, Hami et al., investigated the effects of maternal diabetes during pregnancy on the expression of synaptophysin in the cerebellum of pups (102). Their survey revealed that GDM adversely affected synaptophysin gene expression in the offspring’s cerebellum. The study mentioned earlier stated that most subjects’ blood glucose control during pregnancy could be normalized to these adverse effects. On the other hand, the researcher mentioned above suggested that gestational hyperglycemia accompanied by neonatal hyperinsulinemia was able to decrease the synaptogenesis process in the pup’s cerebellar cortex.

## In conclusion 

The occurrence of some disorders in offspring that are born to diabetic mothers is more common in comparison to offspring of non-diabetic mothers. The significant role that GDM plays in causing the disorders mentioned above has prompted considerable efforts from clinical and experimental researchers. The final aim of all studies was to find the biomarkers or factors that may enable measures to be taken in GDM. GDM, as a neurotoxic agent, impaired cerebellar development and could be a cause for the behavioral, functional, and structural anomalies observed in pups of diabetic mothers. Based on the literature review, most studies have pointed out that insulin administration in patients with GDM decreased the cellular and molecular alterations induced by GDM in the developing cerebellum. Undoubtedly, screening strategies for all pregnant women are necessary. 

## Author’s Contribution

MJ.SB: Designing the project, collecting articles and preparing the manuscript. 

P.CF and E.AI: Revising manuscripts and giving comments to improve the manuscript. MG: collecting data and appropriate articles, editing draft

## Conflict of Interest

The authors declare no conflict of interest
